# Buffalo Academy of Medicine

**Published:** 1899-05

**Authors:** Thomas. F. Dwyer


### Society Proceedings.

### BUFFALO ACADEMY OF MEDICINE.

Reported by THOMAS. F. DWYER, M. D., Secretary.

THE regular quarterly meeting was held at the Academy parlors, Tuesday evening, March 21, 1899. In the absence of the president, Dr. Roswell Park, the meeting was called to order at 9 o'clock p. m., by vice-president Dr. A. L. Benedict, chairman of the section of pathology.

The minutes of the last quarterly meeting, as read by the secretary, were approved.

The nominations of officers resulted as follows: For president, Dr. Matthew D. Mann; secretary, Dr. Thomas F. Dwyer; treasurer, Dr. Eugene A. Smith, Dr. Nelson G. Russell; trustee, Dr. Stephen Y. Howell, Dr. Ludwig Schroeter.

Dr. A. L. Benedict was called away and vice-president, Dr. William G. Ring, chairman of the section of medicine, occupied the chair during the remainder of the meeting.

The council recommended the election of Drs. Charles F. Durand and M. Axford for resident membership, who were separately balloted for and duly elected Fellows of the Academy. The section of pathology provided the following program: Tuberculous infections in the walls of the blood-vessels and the production of miliary tuberculosis, by Dr. H. R. Gaylord. Discussion by Drs. Rochester, Van Peyma, Howell and Blaauw.

Report of case of cholecystitis and presentation of specimen, by Dr. Sidney A. Dunham. Attendance, 36. Meeting adjourned at 10.30 p. m.

